# Impact of socio‐economic deprivation on endometrial cancer survival in the North West of England: a prospective database analysis

**DOI:** 10.1111/1471-0528.16618

**Published:** 2021-01-11

**Authors:** K Njoku, CE Barr, L Hotchkies, N Quille, YL Wan, EJ Crosbie

**Affiliations:** ^1^ Division of Cancer Sciences School of Medical Sciences Faculty of Biology, Medicine and Health St Mary’s Hospital University of Manchester Manchester UK; ^2^ Division of Cancer Sciences Faculty of Biology, Medicine and Health Stoller Biomarker Discovery Centre University of Manchester Manchester UK; ^3^ Department of Obstetrics and Gynaecology Manchester University NHS Foundation Trust Manchester Academic Health Science Centre Manchester UK

**Keywords:** Endometrial cancer, prognosis, recurrence, socio‐economic deprivation, survival

## Abstract

**Objective:**

To assess the impact of socio‐economic deprivation on endometrial cancer survival.

**Design:**

Single‐centre prospective database study.

**Setting:**

North West England.

**Population:**

Women with endometrial cancer treated between 2010 and 2015.

**Methods:**

Areal‐level socio‐economic status, using the English indices of multiple deprivation from residential postcodes, was analysed in relation to survival using Kaplan–Meier estimation and multivariable Cox regression.

**Main outcome measures:**

Overall survival, cancer‐specific survival and patterns and rates of recurrence.

**Results:**

A total of 539 women, with a median age of 66 years (interquartile range, IQR 56–73 years) and a body mass index (BMI) of 32 kg/m^2^ (IQR 26–39 kg/m^2^), were included in the analysis. Women in the most deprived social group were younger (median 64 years, IQR 55–72 years) and more obese (median 34 kg/m^2^, IQR 28–42 kg/m^2^) than women in the least deprived group (median age 68 years, IQR 60–74 years; BMI 29 kg/m^2^, IQR 25–36 kg/m^2^; *P* = 0.002 and <0.001, respectively). There were no differences in endometrial cancer type, stage or grade between social groups. There was no difference in recurrence rates, however, women in the middle and most deprived social groups were more likely to present with distant/metastatic recurrence (80.6 and 79.2%, respectively) than women in the least deprived group (43.5%, *P* < 0.001). Women in the middle and most deprived groups had a two‐fold (adjusted hazard ratio, HR = 2.00, 95% CI 1.07–3.73, *P* = 0.030) and 53% (adjusted HR = 1.53, 95% CI 0.77–3.04, *P* = 0.221) increase in cancer‐specific mortality compared with women in the least deprived group. There were no differences in overall survival.

**Conclusions:**

We found that socio‐economically deprived women with endometrial cancer were more likely to develop fatal recurrence. Larger studies are needed to confirm these findings and to identify modifiable contributing factors.

**Tweetable abstract:**

Socio‐economic deprivation is linked to an increased risk of death from endometrial cancer in the North West of England.

## Tweetable abstract

Socio‐economic deprivation is linked to an increased risk of death from endometrial cancer in the North West of England.

## Introduction

Endometrial cancer is the most common gynaecological malignancy in the developed world, and its incidence is rising.^1^ Although most women with endometrial cancer are diagnosed at an early stage when curative treatment is likely, a significant minority present with advanced disease and face a very poor prognosis.^1^, ^2^ In the UK, over 20% of women with endometrial cancer die within 5 years of diagnosis.^3^ Identifying factors that influence survival is important to improve outcomes from this disease.

Socio‐economic status (SES) is a strong, consistent and reliable determinant of life expectancy and health‐related quality of life.^4^, ^5^ Social gradients in health outcomes relate to differences in nutrition, physical activity, educational attainment, health‐related behaviours and so on.^6^, ^7^, ^8^ Neighbourhoods can significantly influence health outcomes through the physical environment (e.g. air and water quality, residential proximity to environmental hazards) and by affecting the availability and quality of educational, medical, employment and transportation services.^7^, ^8^ Collectively, these factors shape a resident’s opportunity to make a living, influence their health‐seeking behaviour and impact their ability to access medical care.^6^, ^9^ The availability of nutritious food and safe places to exercise vary widely between neighbourhoods and are directly linked to body mass index (BMI), cardiovascular fitness and the risk of many diseases, including endometrial cancer.^8^, ^10^

Disparities in survival by social class have been described for most adult cancers, with patients from higher social classes having better survival outcomes than patients who are socio‐economically deprived.^11^, ^12^ Few studies have examined the role of socio‐economic deprivation on survival from endometrial cancer, however.^13^ A recent systematic review identified nine studies, only two of which were based in the UK, with both failing to account for clinical factors that may confound or explain the prognostic effects of SES.^14^, ^15^ Obesity (BMI ≥ 30 kg/m^2^), age at diagnosis, the presence of comorbidities, and disease stage, grade and histological subtype all influence outcomes following cancer treatment.^16^, ^17^ The interactions between these factors, SES and survival in the context of endometrial cancer are unclear and warrant further study.

Using data from a prospectively maintained database, we examined whether endometrial cancer survival outcomes vary by areal‐level (neighbourhood) SES in the North West of England, a region known to have high levels of socio‐economic deprivation and persistently poor health outcomes when compared with the rest of England. Furthermore, we assessed for the presence of interactions between social class and endometrial cancer clinical and prognostic factors.

## Methods

### Study population

Women with endometrial cancer treated between 2010 and 2015 at St Mary’s Hospital, Manchester, who had consented for their pseudo‐anonymised data, including clinical outcomes, to be used for future research, were eligible for inclusion. Sociodemographic (age at diagnosis, residential postcode at diagnosis) and clinicopathological data (BMI, comorbidities, histological subtype, tumour stage and grade) were recorded at baseline. Age at diagnosis was categorised as <50, 50–70 and >70 years and women were considered underweight (BMI < 18.5 kg/m^2^), normal weight (BMI from 18.5 to <25 kg/m^2^), overweight (BMI from 25 to <30 kg/m^2^) or obese (BMI ≥ 30 kg/m^2^). Endometrial cancers were classified according to histological subtype (endometrioid, serous, clear cell, carcinosarcoma) using confirmatory immunohistochemistry, as necessary, by two specialist gynaecological pathologists, and these data were then collapsed into Bohkman’s dichotomous groupings (types 1 and 2).^18^ The revised FIGO (International Federation of Gynecology and Obstetrics) 2009 classification was used for disease staging.^19^ Most women underwent total hysterectomy and bilateral salpingo‐oophorectomy, with or without adjuvant treatment, in accordance with international guidance.^14^, ^20^ Primary hormone therapy (with or without adjuvant hysterectomy) was used for grade‐1 stage‐1a endometrial cancer in premenopausal women wishing to preserve their fertility, as well as in women assessed as medically unfit for surgery. A few women had primary palliative radiotherapy. Women were reviewed in follow‐up clinics at 3‐month (for 3 years), 6‐month (for 1 year) and 12‐month intervals for a total of 5 years, or until recurrence or death, whichever was sooner. GPs were contacted to determine current status if the women had been discharged from routine hospital‐based follow‐up or had moved away from the area. Recurrent disease was treated according to national and international guidance. Pelvic recurrence was managed by radiotherapy or surgery, where appropriate, whereas unsalvageable multisite, inoperable pelvic or distant recurrent disease was managed with palliative hormone therapy, chemotherapy or radiotherapy.^14^, ^16^, ^20^ Cause of death was obtained from death certificates.

### Socio‐economic status

St Mary’s Hospital is a regional specialist referral centre for the treatment and management of gynaecological cancers. It treats women with endometrial cancer referred directly from primary care as well as women with high‐risk disease from local cancer units. It serves a large geographical area of over 6000 square miles and a population of approximately 3.2 million, residing in diverse communities that range from dense, heavily populated urban areas to remote and widespread rural communities. Residential postcodes at diagnosis were used to determine the index of multiple deprivation rank and deciles of deprivation. The English indices of deprivation is the official measure of relative deprivation assessment in England, and follows an established framework encompassing a wide range of living conditions, specifically income, employment, health deprivation and disability, education, crime, and barriers to housing and services.^21^ All English neighbourhoods are then ranked according to their level of deprivation, from 1 (most deprived area) to 32 844 (least deprived area). For ease of analysis and to facilitate comparison with other studies, deprivation deciles were collapsed into three social deprivation groups: social group I/least deprived (deciles 7–10), social group II/moderately deprived (deciles 3–6) and social group III/most deprived (deciles 1 and 2).

### Statistical analysis

All study variables were treated as categorical variables except age and BMI, which were also considered as numerical variables. Survival time was defined as the time in months from the date of first treatment to death from any cause or the last day of availability of survival information. Cancer‐specific survival was calculated from the date of first treatment to death from endometrial cancer or last date of survival data and censored on date of death from other causes. Recurrence‐free survival was calculated from the date of primary treatment to the first record of recurrence, death or last day of follow‐up data, whichever was sooner. Where no events were observed during the 10‐year study period, subjects were right censored up to a maximum of 120 months post‐treatment. The survival variable was thus binary, with two levels: dead or censored (for overall and cancer‐specific survival) and recurrence or censored (for recurrence‐free survival). The Kruskal–Wallis test was used to investigate differences in median age and BMI at diagnosis between social groups, whereas the chi‐square test was used to test associations between categorical variables. The Kaplan–Meier method was used to compute 1‐, 3‐ and 5‐year survival rates and the log‐rank test was used to assess for survival differences between groups. Cox regression analysis was used for the multivariable modelling of the association between SES and survival while adjusting for confounding and effect modifications. Hazard ratios (HRs) with 95% confidence intervals (95% CIs) were reported for both univariable and multivariable analyses. Interactions were tested within the regression framework and confounding was assessed by changes in SES coefficients following the introduction of other covariates in the regression model. The likelihood ratio test was used to assess for nesting effects. The assumption of proportional hazards was assessed and met for all models. *P* < 0.05 was considered statistically significant. The statistical package stata 16.0 (https://www.stata.com) was used for all analyses.

### Funding

Cancer Research UK (CRUK) Manchester Cancer Research Centre Clinical Research Fellowship (C147/A25254) and the National Institute of Health Research Manchester Biomedical Research Centre (IS‐BRC‐1215‐20007).

## Results

### Descriptive characteristics of the study population

The study population comprised 539 women with histologically confirmed endometrial cancer (Table 1). Their median age and BMI were 66 years (interquartile range, IQR, 56–73 years) and 32 kg/m^2^ (IQR 26–39 kg/m^2^), respectively. Most women were 50–70 years of age (54.9%), overweight or obese (83.5%), with low‐grade (67.7% grade I/II), early‐stage (74.8% stage 1), endometrioid (75.0%) endometrial cancer. Treatment was primary hysterectomy in 473 women (87.8%) and hormone therapy for fertility‐sparing and surgical fitness reasons in 23 (4.3%) and 40 (7.4%) women, respectively. Twelve women (52.2%) who had primary fertility‐sparing hormonal treatment received a hysterectomy during the study period.

**Table 1:** Socio‐demographic characteristics of study participants

Variable	*n* (% total)
Age at diagnosis	Median age 66 years (IQR 56–73 years)
<50 years	61 (11.3%)
50–70 years	296 (54.9%)
>70 years	182 (33.8%)
Body mass index (BMI, kg/m^2^)	Median BMI 32 kg/m^2^ (IQR 26–39 kg/m^2^)
Underweight	6 (1.1%)
Normal weight	83 (15.4%)
Overweight	128 (23.8%)
Obese	322 (59.7%)
Grade of endometrial cancer	
1	243 (45.1%)
2	122 (22.6%)
3	174 (32.3%)
Stage of endometrial cancer	
I	403 (74.9%)
II	56 (10.4%)
III	71 (13.0%)
IV	9 (1.7%)
Histology	
Endometrioid	404 (75.0%)
Non‐endometrioid	135 (25.0%)
Bohkman’s category	
Type 1	364 (67.5%)
Type 2	175 (32.5%)
Lymphovascular invasion (*n* = 536)	
No	382 (71.3%)
Yes	154 (28.7%)
Depth of myometrial invasion	
<50%	346 (64.2%)
≥50%	193 (35.8%)
Social deprivation group (*n* = 532)	
Social group I (least deprived)	147 (27.6%)
Social group II (middle group)	186 (35.0%)
Social group III (most deprived)	199 (37.4%)
History of diabetes mellitus (*n* = 535)	
Yes	108 (20.2%)
No	427 (79.8%)
Primary treatment	
Surgery	473 (87.8%)
Hormonal (fertility‐sparing reasons)	23 (4.3%)
Hormonal (not fit for surgery)	40 (7.4%)
Radiotherapy	3 (0.6%)
Adjuvant treatment	
Yes	240 (44.5%)
No	299 (55.5%)
Recurrence	
Yes	78 (14.5%)
No	460 (85.5%)
Survival status at end of follow‐up	
Alive	429 (79.6%)
Cancer‐specific mortality	76 (14.1%)
Non‐cancer related mortality	34 (6.3%)
Total	539 (100%)

The modal social group was social group III (most deprived) and accounted for 37.4% of the studied population. Postcodes were unmatched for seven women (1.3% of cases). There was a significant trend in age and BMI distributions across the ordered social group categories (*P* < 0.001 for trend). Women in the most deprived social group were younger and more obese than women in the middle and least deprived groups, respectively (Table S1). Women with type‐2 diabetes mellitus were more likely to be obese than women without diabetes (74.1 vs 56.8%, respectively; *P* = 0.003). The presence of lymphovascular space invasion (LVSI) differed by social group, being reported in 24.5% of the least deprived group, 35.3% of the middle group and 25.3% of the most deprived group, respectively (χ^2^ = 6.39, *P* = 0.04). There was no evidence of an association between social groupings and diabetic status (15.0% social group I, 18.9% social group II, 25.0% social group III; *P* = 0.064), Bokhman’s type‐1 grouping (61.9% social group I, 67.7% social group II, 71.9% social group III; *P* = 0.147), FIGO stage 1 (73.5% social group I, 74.6% social group II, 76.4% social group III; *P* = 0.410), grade‐1 disease (39.5% social group I, 44.1% social group II, 49.8% social group III; *P* = 0.350), ≥50% depth of myometrial invasion (42.2% social group I, 33.3% social group II, 33.2% social group III; *P* = 0.158) or treatment received in either primary (surgery, 89.1% social group I, 88.7% social group II, 85.9% social group III; *P* = 0.756) or adjuvant (49.7% social group I, 46.0% social group II, 39.7% social group III; *P* = 0.166) settings (Table S1). Over the study period and irrespective of social deprivation status, 78 women (14.5%) relapsed, 110 (20.4%) died, eight (1.5%) were lost to follow‐up and the remainder were alive as of 31 March 2020.

### Kaplan–Meier survival estimation and Cox regression analysis

Crude survival estimates and unadjusted hazard ratios based on univariable analysis are presented in Table 2 and Cox regression analyses are presented in Table 3. The median follow‐up was 39 months (range 1–120 months). The overall survival rates for the study cohort were 95% (95% CI 92–96%) at 12 months, 85% (95% CI 81–88%) at 36 months and 76% (95% CI 71–80%) at 60 months. There was no difference in overall survival according to areal‐level socio‐economic grouping in the univariable analysis (Table 2).

**Table 2:** Overall survival rates at 1, 3 and 5 years, and crude hazard ratios and 95% confidence intervals, by demographic and clinical predictors

Variable	1‐year survival % (95% CI)	3‐year survival % (95% CI)	5‐year survival % (95% CI)	Hazard ratio (95% CI)	*P*
Age					
<50 years	100	97 (82–100)	97 (82–100)	1.00	
50–70 years	96 (94–98)	89 (84–92)	80 (74–86)	9.66 (1.33–70.0)	0.025
>70 years	89 (83–93)	72 (64–79)	64 (54–72)	24.33 (3.37–175.8)	0.002
Stage of endometrial cancer					
Early stage (I/II)	97 (95–98)	88 (84–91)	81 (76–85)	1.00	
Late stage (III/IV)	78 (67–86)	64 (52–74)	50 (34–64)	3.17 (2.11–4.76)	<0.001
Grade					
1	97 (94–99)	91 (86–95)	88 (82–92)	1.00	
2	97 (92–99)	91 (83–95)	83 (72–90)	1.51 (0.83–2.73)	0.175
3	88 (82–92)	71 (63–77)	59 (49–67)	3.71 (2.32–5.93)	<0.001
Bokhman’s type					
Type 1	98 (95–99)	91 (88–94)	84 (79–89)	1.00	
Type 2	88 (82–92)	71 (63–77)	59 (49–67)	3.04 (2.08–4.45)	<0.001
Lymphovascular space invasion (LVSI)					
No	96 (94–98)	89 (85–92)	81 (76–86)	1.00	
Yes	90 (84–94)	73 (65–80)	62 (51–71)	2.28 (1.56–3.31)	<0.001
Depth of myometrial invasion					
<50%	96 (94–98)	88 (84–92)	80 (74–85)	1.00	
≥50%	91 (86–95)	78 (70–83)	68 (59–76)	1.79 (1.23–2.61)	0.002
History of diabetes mellitus					
No	95 (92–97)	87 (83–90)	80 (75–84)	1.00	
Yes	92 (85–96)	73 (63–81)	62 (50–72)	1.91 (1.27–2.86)	<0.001
Body mass index categories					
Normal weight	99 (91–100)	93 (85–97)	82 (67–90)	1.00	
Overweight	96 (91–98)	81 (72–87)	77 (67–84)	1.73 (0.88–3.38)	0.110
Obese	93 (90–96)	84 (79–88)	75 (68–81)	1.66 (0.90–3.08)	0.107
Social deprivation groups					
Least deprived	94 (88–97)	84 (77–89)	73 (62–81)	1.00	
Middle group	94 (90–97)	82 (75–87)	70 (60–78)	1.16 (0.73–1.84)	0.523
Most deprived	95 (91–97)	88 (82–92)	83 (75–88)	0.75 (0.46–1.24)	0.267

**Table 3:** Cox regression analysis of social class and survival outcomes in endometrial cancer, adjusted for age, body mass index (BMI), history of diabetes mellitus, Bokhman’s group, FIGO stage, lymphovascular space invasion (LVSI), depth of myometrial invasion and treatment received

Social class	Hazard ratio (95% CI)	*P* value
Overall mortality		
Least deprived	1.00	
Middle group	1.31 (0.80–2.13)	0.281
Most deprived	1.08 (0.63–1.86)	0.766
Cancer‐specific mortality		
Least deprived	1.00	
Middle group	2.00 (1.07–3.73)	0.030
Most deprived	1.53 (0.77–3.04)	0.221
Recurrence		
Least deprived	1.00	
Middle group	1.04 (0.59–1.81)	0.903
Most deprived	0.96 (0.52–1.77)	0.903

Overall survival was higher in women diagnosed with early‐stage, low‐grade endometrial cancer (Table 2). There was a 7% increased risk of death per unit increase in age at diagnosis (HR = 1.07, 95% CI 1.05–1.09, *P* < 0.0001). There was no evidence of an effect of BMI on overall survival (HR per unit increase in BMI = 0.99, 95% CI 0.97–1.01, *P* = 0.57); however, women with diabetes mellitus had a 91% increased risk of death compared with women without diabetes mellitus (HR = 1.91, 95% CI 1.27–2.86, *P* = 0.002). Women with LVSI had a two‐fold higher mortality risk (HR = 2.28, 95% CI 1.56–3.31, *P* < 0.001) compared with women with no LVSI, whereas women with ≥50% myometrial invasion had a 79% higher risk of death (HR = 1.79, 95% CI 1.23–2.61). Compared with women whose primary treatment was surgery, the women who were deemed unfit for hysterectomy (and who received hormonal treatment) had a two‐fold higher risk of death (HR = 2.04, 95% CI 1.05–3.94, *P* = 0.034), mostly from causes unrelated to cancer (7/10, 70%), whereas women who had palliative radiotherapy had an eight‐fold higher risk of death from endometrial cancer (HR = 8.16, 95% CI 1.98–33.6, *P* = 0.004).

Following adjustment for age, BMI, diabetes status, Bokhman’s type, FIGO stage, LVSI, depth of myometrial invasion and treatment received, there was no effect of SES on all‐cause mortality (Table 3). Only age at diagnosis (HR = 1.06, *P* < 0.001), FIGO stage (HR = 2.3, *P* = 0.001), Bokhman’s type (HR = 2.46, *P* < 0.001), LVSI (HR = 1.62, *P* = 0.03) and treatment received (HR = 3.52, *P* = 0.004) were associated with overall survival in the multivariable analysis.

### Socio‐economic deprivation and cancer‐specific survival

Of the 110 deaths, 76 (69.1%) were a result of endometrial cancer whereas the remaining 34 (30.9%) were a result of other causes, including cardiac and respiratory conditions as well as other malignancies and life events. Cancer‐specific survival for the whole cohort was 96% (95% CI 94–97%) at 12 months, 89% (95% CI 86–91%) at 36 months and 82% (95% CI 77–86%) at 60 months. Univariable analysis indicated that there was no evidence of an effect of SES on endometrial cancer‐specific survival in social group II (unadjusted HR = 1.53, 95% CI 0.85–2.73, *P* = 0.155) or social group III (unadjusted HR = 0.94, 95% CI 0.50–1.77, *P* = 0.842). After adjusting for age, BMI, diabetes status, Bokhman’s type, FIGO stage, LVSI, depth of myometrial invasion and treatment received, however, women in the middle social group had a two‐fold increased risk (HR = 2.00, 95% CI 1.07–3.73, *P* = 0.030) and women in the most deprived group had a 53% increased risk (HR = 1.53, 95% CI 0.77–3.04, *P* = 0.221) of cancer‐specific death, compared with women in the least deprived group, respectively (Table 3). As expected, age (HR = 1.05 per year increase, *P* < 0.001), Bokhman’s type (HR = 3.99, *P* < 0.001), FIGO stage (HR = 3.29, *P* < 0.001) and LVSI (HR = 2.25, *P* = 0.003) were all associated with cancer‐specific survival in the multivariable analysis.

### Socio‐economic deprivation and disease recurrence

Over the study period, 78 women (14.5%) relapsed with a median time to recurrence of 13 months (range 1–54 months). The recurrence‐free survival rate was 93% (95% CI 90–95%) at 12 months, 83% (95% CI 79–86%) at 36 months and 80% (95% CI 75–84%) at 60 months. After adjusting for confounding factors, there was a 4% increased risk of recurrence per year increase in age (HR = 1.04, *P* = 0.002). Bokhman’s group (HR = 3.27, *P* < 0.001), FIGO stage (HR = 2.60, *P* < 0.001) and LVSI (HR = 2.15, *P* = 0.004) were also significant predictors of recurrence in the multivariable analysis. Overall, 23/147 (15.6%), 30/185 (16.2%) and 24/199 (12.1%) women in the least, middle and most deprived social groups relapsed. The corresponding 5‐year recurrence‐free survival rates were 76% (95% CI 65–84%), 77% (95% CI 68–84%) and 85% (95% CI 78–89%) in the least, middle and most deprived social groups, respectively. There was no evidence of an association between recurrence rates and social class in either univariable or multivariable analyses (Tables 3 and 4); however, women in the middle and most deprived social groups were more likely to present with distant recurrent disease (80.6% and 79.2%, respectively) than women in the least deprived group (43.5%, *P* < 0.001). In addition, women in the least deprived social group presented on average 4 months earlier at relapse. The median time between recurrence and death was 7.5 months (range 1–54 months), 6.8 months (range 1–50 months) and 3.3 months (range 1–30 months) for the least, middle and most deprived social groups, respectively. Although 39% (9/23) of the least deprived women who relapsed were salvaged (i.e. did not succumb to their disease following second‐line treatment), this was the case in only 13% (4/30) and 33% (8/24) of women in the middle and most deprived social groups (*P* < 0.001), respectively.

**Table 4:** Recurrence‐free survival rates at 1, 3 and 5 years, and crude recurrence hazard ratios and 95% confidence intervals, by socio‐economic status and demographic and clinical predictors

Variable	1‐year survival % (95% CI)	3‐ year survival % (95% CI)	5‐year survival % (95% CI)	Hazard ratio (95% CI)	*P*
Age					
<50 years	100	98 (87, 100)	98 (87–100)	1.00	
50–70 years	92 (87–95)	83 (76–87)	81 (75–86)	9.05 (1.25–65.7)	0.029
>70 years	92 (86–95)	78 (69–84)	70 (58–79)	13.42 (1.83–98.3)	0.011
Stage of endometrial cancer					
Early stage (I/II)	96 (94–98)	88 (84–91)	85 (80–89)	1.00	
Late stage (III/IV)	77 (65–85)	56 (43–68)	52 (37–65)	4.65 (2.93–7.38)	<0.001
Grade					
1	99 (96–100)	95 (90–97)	94 (88–97)	1.00	
2	97 (92–99)	86 (76–92)	80 (68–87)	3.22 (1.42–7.28)	0.005
3	83 (76–88)	66 (58–74)	63 (54–71)	8.46 (4.16–17.18)	<0.001
Bokhman’s group					
Type 1	98 (96–99)	91 (87–94)	88 (83–92)	1.00	
Type 2	82 (75–87)	66 (57–73)	63 (54–71)	4.81 (3.00–7.70)	<0.001
Lymphovascular space invasion (LVSI)					
No	96 (93–97)	91 (87–93)	88 (83–92)	1.00	
Yes	87 (80–91)	64 (55–72)	59 (49–68)	4.04 (2.57–6.34)	<0.001
Depth of myometrial invasion					
<50%	96 (93–98)	88 (83–91)	85 (80–89)	1.00	
≥50%	88 (82–92)	75 (66–81)	70 (60–77)	2.41 (1.55–3.77)	<0.001
History of diabetes mellitus					
No	94 (91–96)	85 (81–89)	81 (76–86)	1.00	
Yes	89 (81–94)	72 (60–81)	72 (60–81)	1.74 (1.06–2.85)	0.029
Body mass index categories					
Normal weight	97 (90–99)	85 (74–92)	83 (70–90)	1.00	
Overweight	92 (85–96)	81 (71–87)	76 (64–84)	1.45 (0.70–3.01)	0.317
Obese	93 (89–95)	84 (78–88)	81 (75–86)	1.17 (0.60–2.27)	0.643
Social deprivation group					
Least deprived	92 (86–96)	84 (75–89)	76 (65–84)	1.00	
Middle group	94 (89–96)	81 (73–86)	77 (68–84)	1.00 (0.58–1.72)	0.998
Most deprived	93 (88–96)	85 (78–90)	85 (78–90)	0.79 (0.45–1.40)	0.418

## Discussion

### Main findings

In this study, we identified differences in endometrial cancer survival outcomes in the North West of England according to SES. Although overall survival was similar across all socio‐economic groups, after adjusting for potential confounding factors, women from the middle and most deprived socio‐economic groups were more likely to die from endometrial cancer than women from the least deprived group. This may be partly explained by patterns of relapse, with women from the most deprived areas being more likely to present with metastatic and rapidly fatal recurrent disease than women from more affluent neighbourhoods. The association between SES and endometrial cancer survival was not linear, with women in the middle social group having poorer outcomes than both the most and least deprived social groups. The relatively small number of survival events across the three socio‐economic groups limits the certainty of our conclusions, and larger studies are now needed to confirm our findings and to identify possible explanations.

### Strengths and limitations

We analysed survival outcomes from a large number of endometrial cancer patients recruited to our prospectively maintained database. Most women were recruited to population‐based studies that posed no restriction according to tumour factors, thereby alleviating any concerns about potential selection bias. Complete demographic and clinicopathological data enabled a robust adjustment for potential confounding factors. All women were treated through the publicly funded UK National Health Service, which minimised any differences in treatment related to the ability to pay. The use of an areal measure of SES, specifically the English indices of multiple deprivation, captured the broader issues of neighbourhood and ecological contextual effects of SES on survival outcomes.^22^ This measure is prone to so‐called ecological fallacy, however, wherein inferences are made based on aggregate group data that may not accurately align with an individual’s SES.^23^ We did not have data relating to individual circumstances, like educational status and income, which reduce the risk of misclassification by area‐level indicators.^24^ The lack of complete data on ethnicity precluded an assessment of ethnic disparities on endometrial cancer survival. We collapsed deprivation deciles into three socio‐economic groups based on the distribution of cases; however, this may inadvertently misclassify women relative to the overall English population. Molecular subgroup classification data based on the four prognostic categories of The Cancer Genome Atlas (TCGA) were not available for this study, and may introduce residual confounding and an over‐ or underestimation of survival outcomes.^25^ Women may change residential location over time,^26^ although most of the women in this study did not move during follow‐up. Although we describe outcomes from a large cohort of endometrial cancer patients, the generally good prognosis and consequent low event rate affects the reliability of our conclusions. The single‐centre nature of this study is a further limitation of our work, as we cannot necessarily extrapolate our findings to other centres, countries or healthcare settings.

### Interpretation

Few studies have examined the role of SES on endometrial cancer survival outcomes,^13^ and only three have been based in the UK.^14^, ^15^, ^27^ Gildea and colleagues found no association between 30‐day postoperative mortality and income deprivation, but failed to assess long‐term outcomes.^15^ The National Cancer Intelligence Network reported an association between income deprivation and endometrial cancer survival in England, with women from socially deprived backgrounds having a higher overall mortality compared with women from less deprived backgrounds.^14^ This study was unable to adjust for important prognostic factors like age, BMI and comorbidities, however, that may explain some of the observed associations. A recent study by Donkers and colleagues, involving 688 women with endometrial cancer, found no social gradient in survival outcomes after adjusting for confounding factors.^27^ Interestingly, women from more affluent communities had a higher rate of recurrence than women from deprived communities, but this did not translate into an increase in cancer‐specific deaths. The least deprived social group was under‐represented, comprising just 5% of their total cohort compared with 28% of our cohort. This may relate to differences in the way the two studies collapsed the deprivation deciles into three social groups, as the North West of England has some of the most deprived neighbourhoods in England. Despite inconsistent findings from UK studies, analyses from the USA, Denmark and Japan all show an association between socio‐economic deprivation and poor endometrial cancer survival outcomes, with race serving as a proxy for SES in many studies.^28^, ^29^, ^30^, ^31^, ^34^

Socio‐economic disparities in cancer survival may be related to patient (age, obesity, comorbidities, health‐seeking behaviours), tumour (cancer stage and grade, tumour biology) or healthcare factors (access to health care, variation in quality of care).^35^ In our study, women from the most deprived neighbourhoods were more obese than women from less deprived neighbourhoods, in keeping with the literature.^35^, ^36^, ^37^ Obesity‐driven endometrial cancer is usually low‐grade, early‐stage disease with good survival outcomes when compared with aggressive, non‐endometrioid histological subtypes.^38^, ^39^ Despite advantages in tumour biology, however, obesity is linked to unfavourable survival outcomes through a high prevalence of related comorbid health conditions.^40^ This not only increases the risk of death from other causes but is also linked to cancer‐specific death related to treatment factors. For example, women with class‐III obesity are less likely to be offered surgery, have a higher risk of perioperative complications and are liable to receive suboptimal doses of chemotherapy as a result of dose restriction.^41^, ^42^, ^43^, ^44^ Type‐2 diabetes mellitus was more common in women who were obese compared with women who were not obese (25 vs 10.8%, respectively), and was associated with a higher risk of death in this study and in others.^45^ Neither obesity nor diabetes status mediated the link between SES and endometrial cancer‐specific survival, however, as the socio‐economic gradient in survival outcomes was only significant after adjusting for these variables.

Endometrial cancer has a generally good prognosis because most women present following the onset of postmenopausal bleeding, when the tumour is confined to the uterus.^46^, ^47^ Stage at diagnosis is the most important prognostic factor for the majority of adult cancers. An advanced stage may reflect a delay in health seeking, aggressive tumour biology and/or a delay in healthcare provision.^11^ There was no association between stage and socio‐economic deprivation in our study and there was minimal evidence for confounding; adjustment for stage did not substantially influence SES hazard ratios. This may be linked to the preponderance of early‐stage disease in our study, with 85% of our cohort diagnosed with stage‐I/II endometrial cancer, irrespective of SES.

Social disparities in cancer survival may also be linked to differences in the type and quality of clinical care offered to patients from different social groups.^12^, ^35^ Adjuvant radiotherapy and/or chemotherapy is recommended for endometrial cancer patients at high risk of progression and systematic variations in adherence to treatment protocols may explain some SES survival effects. When access to good‐quality care is the same, social gradients in outcomes are reduced.^48^ Although universal access to world‐class treatment might be presumed in the non‐private healthcare settings of developed countries like England, it is possible that SES‐related issues in seeking and obtaining care, or in compliance with treatment, contribute to disparities in outcome. We found no difference in primary treatment allocation or recurrence‐free survival related to SES; however, women from more deprived socio‐economic backgrounds were more likely to present with metastatic, rapidly fatal, recurrent disease than women from more affluent backgrounds. Women in the least deprived social group presented on average 4 months earlier at relapse and were three‐fold more likely to develop localised pelvic recurrence amenable to curative surgery. These findings may relate to differential patterns of health‐seeking behaviour at relapse and could be modifiable through educational interventions.

## Conclusion

We found a socio‐economic disparity in cancer‐specific survival amongst women treated for endometrial cancer in the North West of England. Socio‐economically disadvantaged women were more likely to present with fatal relapse than women from less deprived backgrounds. Further research is needed to confirm these findings and to determine whether SES‐related barriers to seeking help for relapse may be contributing. Clinical and public health interventions aimed at improving the health‐related behaviours of deprived women with endometrial cancer may minimise avoidable disparities in survival outcomes.

### Disclosure of interests

None declared. Completed disclosure of interests forms are available to view online as supporting information.

### Contribution to authorship

Supervision and funding acquisition, EJC; conceptualization, KN and EJC; study design, KN and EJC; data extraction, KN, CB, LH, NQ, LW and EJC; statistical analysis, KN; writing, original draft preparation, KN; writing, review and editing, EJC; all authors read and approved the final version for publication.

### Details of ethics approval

This study uses data from women participating in clinical research at St Mary’s Hospital, Manchester, who gave written, informed consent for their pseudo‐anonymised clinical data to be used in future research. The primary research studies were: Metformin (North West Research Ethics Committee, NW REC, 11/NW/0442, approved 19 August 2011), Weight loss (NW REC, 12/NW/0050, approved 23 January 2012), PREMIUM (NW REC, 14/NW/1236, approved 23 September 2014), PROTEC (Cambridge East REC, 15/EE/0063, approved 2 April 2015), PETALS (NRES Committee North West, Lancaster, 15/NW/0733, approved 18 September 2015) and DETECT (NW REC, Greater Manchester, 16/NW/0660, approved 16 September 2016).

### Funding

KN is supported by a Cancer Research UK (CRUK) Manchester Cancer Research Centre Clinical Research Fellowship (C147/A25254) and the Wellcome Trust Manchester Translational Informatics Training Scheme. CEB is supported by a Manchester University NHS Foundation Trust Clinical Research Fellowship. LW is supported by a National Institute for Health Research (NIHR) Academic Clinical Lectureship. EJC is supported by the NIHR Manchester Biomedical Research Centre (IS‐BRC‐1215‐20007).

## Supporting information


**Table S1.** Baseline characteristics stratified by socio‐economic group.

Supplementary Material

Supplementary Material

Supplementary Material

Supplementary Material

Supplementary Material

Supplementary Material

## Data Availability

Data are available following reasonable request to the corresponding author.
