# Supplementary material for: Impact of socio‐economic deprivation on endometrial cancer survival in the North West of England: a prospective database analysis
Source: BJOG. 2021 Jan 11;128(7):1215–24. doi: 10.1111/1471-0528.16618 (PMC8248174; doi:10.1111/1471-0528.16618)
Supplement: Supplementary file 1 — Table S1. Baseline characteristics stratified by socio‐economic group. [file BJO-128-1215-s005.pdf]

Table S1. Baseline characteristics stratified by socioeconomic group

Variable	Social group I (n=147)	Social group II (n=186)	Social group III (n=199)	p-value	Total (n=532)
**Age at diagnosis**	Median 68 years	Median 66 years	Median 64 years	0.002	Median 66 years (IQR 56, 73)
<50 years	11 (7.5%)	18 (9.7%)	31 (15.5%)		60 (11.3%)
50-70 years	78 (53.0%)	99 (53.2%)	115 (57.8%)		292 (54.9%)
>70 years	58 (39.5%)	69 (37.1%)	53 (26.7%)		180 (33.8%)
**Body Mass Index (kg/m^2)**	Median 29kg/m^2	Median 32kg/m^2	Median 34kg/m^2	<0.001	Median 32kg/m^2 (IQR 26, 39)
Underweight	1 (0.7%)	0 (0.0%)	5 (2.5%)		6 (1.1%)
Normal weight	31 (21.1%)	29 (15.6%)	23 (11.5%)		83 (15.6%)
Overweight	44 (29.9%)	48 (25.8%)	35 (17.6%)		127 (23.9%)
Obese	71 (48.3%)	109 (58.6%)	136 (68.3%)		316 (59.4%)
**Grade of EC**					
1	58 (39.5%)	82 (44.1%)	99 (49.8%)	0.350	239 (44.9%)
2	34 (23.1%)	44 (23.7%)	44 (22.1%)		122 (22.9%)
3	55 (37.4%)	60 (32.3%)	56 (28.1%)		171 (32.1%)
**Stage of EC**					
I	108 (73.5%)	138 (74.6%)	152 (76.4%)	0.410	398 (75.0%)
II	13 (8.8%)	20 (10.8%)	22 (11.1%)		55 (10.4%)
III	23 (15.7%)	22 (11.9%)	24 (12.0%)		69 (13.0%)
IV	3 (2.0%)	5 (2.7%)	1 (0.5%)		9 (1.6%)
**Histology**					
Endometrioid	105 (71.4%)	138 (74.2%)	156 (78.4%)	0.319	399 (75.0%)
Non-endometrioid	42 (28.6%)	48 (25.8%)	43 (21.6%)		133 (25.0%)
**Bohkman's category**					
Type 1	91 (61.9%)	126 (67.7%)	143 (71.9%)	0.147	360 (67.7%)
Type 2	56 (38.1%)	60 (32.3%)	56 (28.1%)		172 (32.3%)
**Lymphovascular space invasion**					
No	111 (75.5%)	119 (64.7%)	148 (74.8%)	0.041	378 (71.5%)
Yes	36 (24.5%)	65 (35.3%)	50 (25.3%)		151 (28.5%)

**Depth of myometrial invasion**					
<50%	85 (57.8%)	124 (66.7%)	133 (66.8%)		342 (64.3%)
≥50%	62 (42.2%)	62 (33.3%)	66 (33.2%)	0.158	190 (35.7%)
**History of diabetes mellitus**					
Yes	22 (15.0%)	35 (18.9%)	49 (25.0%)	0.064	106 (20.1%)
No	125 (85.0%)	150 (81.1%)	147 (75.0%)		422 (79.9%)
**Primary treatment**					
Surgery	131 (89.1%)	165 (88.7%)	171 (85.9%)	0.756	467 (87.8%)
Hormonal (Fertility sparing reasons)	4 (2.7%)	7 (3.8%)	11 (5.5%)		22 (4.1%)
Hormonal (Not fit for surgery)	12 (8.2%)	12 (6.5%)	16 (8.0%)		40 (7.5%)
Radiotherapy	0 (0.0%)	2 (1.1%)	1 (0.5%)		3 (0.6%)
**Adjuvant treatment**					
Yes	73 (49.7%)	85 (46.0%)	79 (39.7%)	0.166	237 (44.6%)
No	74 (50.3%)	100 (54.1%)	120 (60.3%)		294 (55.3%)
**Recurrence**					
Yes	23 (15.6%)	30 (16.2%)	24 (12.1%)	0.461	77 (14.5%)
No	124 (84.4%)	155 (83.8%)	175 (87.9%)		454 (85.5%)
**Survival status at end of follow up**					
Alive	117 (79.6%)	139 (74.7%)	168 (84.4%)	0.073	424 (79.7%)
Cancer-specific mortality	17 (11.6%)	35 (18.8%)	22 (11.1%)		74 (13.9%)
Non-cancer related mortality	13 (8.8%)	12 (6.5%)	9 (4.5%)		34 (6.4%)

Footnote: Seven women did not have SES data due to unmatched post-codes and were excluded from the findings presented above.
